# Imported Lassa Fever, Pennsylvania, USA, 2010

**DOI:** 10.3201/eid1610.100774

**Published:** 2010-10

**Authors:** Valerianna Amorosa, Adam MacNeil, Ryan McConnell, Ami Patel, Katherine E. Dillon, Keith Hamilton, Bobbie Rae Erickson, Shelley Campbell, Barbara Knust, Deborah Cannon, David Miller, Craig Manning, Pierre E. Rollin, Stuart T. Nichol

**Affiliations:** Author affiliations: University of Pennsylvania, Philadelphia, Pennsylvania, USA (V. Amorosa, R. McConnell, K.E. Dillon, K. Hamilton);; Philadelphia Veterans Affairs Medical Center, Philadelphia (V. Amorosa);; Centers for Disease Control and Prevention, Atlanta, Georgia, USA (A. MacNeil, A. Patel, B.R. Erickson, S. Campbell, B. Knust, D. Cannon, D. Miller, C. Manning, P.E. Rollin, S.T. Nichol);; Philadelphia Department of Health, Philadelphia (A. Patel)

**Keywords:** viruses, Arenaviridae, zoonoses, Lassa fever, travel, imported infections, dispatch

## Abstract

We report a case of Lassa fever in a US traveler who visited rural Liberia, became ill while in country, sought medical care upon return to the United States, and subsequently had his illness laboratory confirmed. The patient recovered with supportive therapy. No secondary cases occurred.

Lassa fever is a potentially severe viral infection caused by *Lassa virus* (family *Arenaviridae*, genus *Arenavirus*), with an overall case-fatality rate of 1%–2% and a case-fatality rate of 15%–20% for hospitalized patients (*1**,**2*). The virus is endemic to West Africa, with the reservoir host being *Mastomys* spp. rodents (*3*). Person-to-person transmission of Lassa virus can occur through direct exposure to infected blood or secretions, and instances of nosocomial transmission have been documented (*4**,**5*). Primary symptoms of Lassa fever are fever, headache, nausea, diarrhea, sore throat, and myalgia; hemorrhagic signs or deafness may also occur during illness (*1**,**6*).

Because the incubation period ranges from a few days to >2 weeks (*1**,**5*) and many symptoms are nonspecific, the potential exists for human carriage of Lassa virus to areas outside those to which it is endemic, putting travel companions, close contacts, and healthcare providers at risk for secondary infection. Before 2010, five instances of imported Lassa virus were recorded in persons from West Africa to the United States. Although early instances involved sick persons who were airlifted to the United States for diagnosis and treatment (*7**–**9*), the 2 most recent occurrences (1989 and 2004) involved persons who were not identified as potentially infectious until healthcare was sought in the United States (*10**,**11*). Here we report a case in a person who became infected and sick during a trip to Liberia and sought care upon return to the United States.

## The Case

A Liberian man 47 years of age living in the United States traveled to Liberia in January 2010. He arrived in Monrovia, then spent 5 days traveling throughout Nimba County in north-central Liberia, bordering Guinea and Côte d’Ivoire. He reported sleeping nightly in his rural native village in a dwelling infested with rats and recalled several rat carcasses on the bedroom floor. On the day of his departure from Liberia, he developed fever, chills, joint pain of the knees and ankles, anorexia, sore throat, diffuse skin tenderness, and mild shortness of breath; he began taking amoxicillin and chloroquine before departing Liberia.

The patient’s symptoms persisted upon arrival in the United States, prompting him to seek medical attention on day 5 of his illness. When he sought treatment, he had fever of 103°F, pulse of 99 beats/min, respiratory rate of 15 breaths/min, and blood pressure of 120/80 mm Hg (Table A1). His physical examination was notable for posterior cervical adenopathy and a palpable spleen tip (Table). He had no evidence of conjunctival, nasal, or oral petechiae; no skin rashes; and no signs of hemorrhage or other lesions. Initial laboratory data showed leukopenia and thrombocytopenia and minimal transaminase elevations (Table A1). Empiric malaria treatment was initiated upon admission and was subsequently discontinued when *Plasmodium* spp. antigen testing was negative and thick and thin blood smears showed no evident parasitemia. By the next day, mild pharyngitis with slight tonsillar exudates had developed. On the third hospital day, substernal chest pain and profuse watery diarrhea developed. Increasing transaminases and a slight coagulopathy were noted. Lassa fever was considered in the differential diagnosis; contact precautions and, subsequently, airborne precautions were taken. Because of noted clinical improvement, he was not given empiric intravenous ribavirin.

**Table:** Day by day symptoms and clinical information for a man 47 years of age with Lassa fever, Pennsylvania, USA, 2010*

Date	Symptoms	Examination findings	Bowel movements	Clinical information	Clinical action
Jan 13–17	Fevers and chills, sore throat, arthralgias, diffuse abdominal pain	NA	NA	NA	NA
Jan 18	Fevers and chills, sore throat, watery diarrhea, diffuse abdominal pain.	Prominent parotids, posterior cervical lymphadenopathy, slight spleen tip	12		Contact isolation ordered.
Jan 19	Fevers and chills, sore throat, substernal chest pain with inspiration and when lying supine, diarrhea, diffuse abdominal pain. Arthralgias resolve.	Slight tonsillar exudates, slight spleen tip	»18	Tests for *Clostridium difficile*, cryptosporidium, *Giardia* spp., thick and thin blood smears for malaria, Epstein-Barr virus, and respiratory virus panel; all negative	Contact precautions. Blood samples to test for Lassa virus drawn.
Jan 20	Fevers and chills; sore throat; substernal chest pain, worse when lying down; diarrhea. Abdominal pain improving.	Slight tonsillar exudates, slight spleen tip	3	HIV negative	EKG done, ribavirin requested, airborne precautions
Jan 21	Fevers and chills, sore throat, diarrhea. Chest pain resolves. Abdominal pain improving.	Slight spleen tip	5	Stool culture negative	Held off on ribavirin. CDC received specimen.
Jan 22	Fevers and chills, sore throat. Diarrhea resolves. Abdominal pain resolves.	Prominent sternocleidomastoid muscles	NA		Stopped IV fluids.
Jan 23	Fevers and chills, sore throat	Prominent sternocleidomastoid muscles	3, formed	PCR positive for Lassa virus	
Jan 24	Fevers and chills, sore throat	Prominent sternocleidomastoid muscles	NA		
Jan 25	Fevers and chills. Sore throat improves.	Prominent sternocleidomastoid muscles	3, formed		
Jan 26	Fevers and chills. Sore throat improves.	Neck less prominent	NA		
Jan 27	Fevers and chills. Sore throat resolves.	Decreased parotid enlargement and lymphadenopathy	NA		
Jan 28–29	Fevers and chills		NA		
Jan 30–Feb 3	Fever resolves		NA		
*NA, not available; EKG, electrocardiogram; IV, intravenous; CDC, Centers for Disease Control and Prevention.					

On day 5 of hospitalization, Lassa virus was identified by real-time PCR by using samples collected 2 days earlier, and sequencing of the amplified fragment yielded a unique sequence similar to sequences from previous Lassa virus isolates from Liberia. Subsequent samples confirmed Lassa fever diagnosis on the basis of real-time PCR, viral culture, and serology (Table).

The patient’s fever resolved by day 16 of his illness. After 2 successive negative blood real-time PCR results, he was discharged from the hospital on day 21 of his illness with instructions to avoid unprotected sexual intercourse for 2 months. No hearing abnormalities were noted at the time of discharge or during telephone conversations 2 weeks and 2 months later.

A contact investigation was undertaken by the hospital and local, state, federal, and international health agencies. Exposed persons were identified as any persons who potentially came into contact with the patient or his body fluids during his illness. Because no contacts had direct exposure to body fluids (other than the patient’s wife in Africa with whom he had sexual intercourse before becoming ill and who remained well, according to telephone follow-up with the patient), no patient contacts were considered high risk for secondary transmission (*10*). In total, 140 persons, including the patient’s family in the United States, co-workers, and hospital workers who had contact with him (but did not have direct contact with bodily fluids) were identified as low-risk contacts. Health communication materials were developed on the basis of previous Lassa fever contact tracing activities (*10*). All hospital and community contacts were provided a Lassa fever fact sheet and asked to seek medical consultation if fever or other signs and symptoms of Lassa fever appeared. Upon completion of 21 days of follow-up, no secondary cases were identified.

## Conclusions

The spectrum of Lassa fever can run from asymptomatic seroconversion to severe hemorrhagic fever with multiple organ failure and death (*1**,**2**,**6*). Factors supporting the diagnosis of Lassa fever in returning travelers include relevant epidemiologic exposure (travel to rural West Africa), signs and symptoms consistent with Lassa fever, and the absence of other infectious agents that can account for the illness. Although this patient did not seek treatment for hemorrhagic signs, his fever, pharyngitis, chest pain, and diarrhea (*1**,**6*), as well as thrombocytopenia and elevated transaminases, were consistent with Lassa fever (*6**,**12*). Early institution of ribavirin can dramatically decrease death rates among patients with severe Lassa fever if given within the first 6 days of illness (*12*); therefore, empiric ribavirin should be considered for an ill patient suspected of having Lassa fever.

As in this case, early suspicion of Lassa fever should prompt isolation measures to avoid secondary transmission; laboratory testing should be limited to essential tests, and all laboratory specimens should be handled with appropriate biosafety precautions to avoid aerosolizing the virus (*13*). Experience in regions where Lassa virus is endemic suggests human-to-human transmission occurs through direct contact with blood and body fluids or large-particle inhalation; transmission through viral aerosolization is not seen; and generally, when universal precautions are undertaken, transmission is unlikely (*14*). Nonetheless, aerosol in addition to contact precautions were undertaken once Lassa fever was suspected, given the theoretical potential for acquiring infection through inhalation of airborne virus from respiratory secretions or, in this case, copious diarrhea.

The patient described in this report represents the sixth known occurrence of Lassa fever imported to the United States. Clinicians treating recent travelers to West Africa who are febrile should obtain detailed histories from patients to determine whether they have traveled into rural areas in which the potential for exposure to rodents exists. The symptoms, signs, and laboratory abnormalities of Lassa fever are nonspecific and can overlap with other tropical infections. Therefore, efforts should be made to promptly diagnose or rule out other infectious agents in a patient who has the appropriate travel and exposure history so that further diagnostic studies and empiric therapy with ribavirin can be undertaken rapidly. Moreover, as soon as Lassa fever is suspected, patients and their specimens should be handled with adequate precautions, the local health department and Centers for Disease Control and Prevention should be notified, and specimens should be sent for specific diagnostic testing.

## Appendix

**Table A1:** Timeline of illness for a patient with Lassa fever, Pennsylvania, USA, 2010

Test results	Jan 13–17†	18 Jan 18‡	Jan 19§	Jan 20	Jan 21	Jan 22	Jan 23	Jan 24	Jan 25	Jan 26	Jan 27	Jan 28–29	Jan 30–Feb 3
Hospital laboratory testing													
Highest temperature	101.0 °F (Jan 17)	103.0°F	103.3°F	103.2°F	101.0°F	101.8°F	100.4°F	99.2°F	101.0°F	101.0°F	101.0°F	Low 100s°F	Low 99s°F
Pulse	99 (Jan 17)	80s	90s	81–103 (EKG normal)	100–110s	100–110	98–100	90s	94–114	91–102	85–110	84–100	ND
Leukocytes	2.7	3	4.7	5.5	5.1	ND	ND	6.5 (normal diff)	ND	ND	ND	ND	ND
Hemoglobin	13.5	13.9	13.7	14	13.9	ND	ND	13.2	ND	ND	ND	ND	ND
Platelets	53	54	61	74	89	ND	ND	236	ND	ND	ND	ND	ND
Sodium	133	134	131	136	132	136	ND	137	ND	ND	ND	ND	ND
Aspartate aminotranferase	43	ND	ND	194	161	121	ND	191	ND	ND	92	ND	ND
Alamine aminotransferase	28	ND	ND	140	153	134	ND	185	ND	ND	161	ND	ND
Alkaline phosphatase	50	ND	ND	44	ND	ND	ND	96	ND	ND	95	ND	ND
Total bilirubin	0.4	ND	ND	0.7	ND	ND	ND	ND	ND	ND	ND	ND	ND
International normalized ratio	ND	ND	1.1	1	ND	ND	ND	ND	ND	ND	ND	ND	ND
Partial thromboplastin time	ND	ND	38.6	30	ND	ND	ND	ND	ND	ND	ND	ND	ND
Culture	Blood 2× (−), Urine (−)	Blood 2× (−), Stool (−)	Blood 2× (−)	Blood 2× (−), *Vibrio* spp. (−), Stool (−)	Blood 2× (−)								
Diagnostic testing for Lassa virus													
Antigen	NA	NA	−	−	−	−	−	−	−	−	NA	−	−
IgG	NA	NA	−	−	−	−	−	−	−	−	NA	−	−
IgM	NA	NA	−	−	−	−	400	1,600	**1,600**	6,400	NA	6,400	6,400
Real-time PCR	NA	NA	+	+	+	+	+	+	+	+	NA	+	+/−¶
Isolation	NA	NA	+	+	+	+	+	+	+	+	NA	+	−

*EKG, electrocardiogram; ND, not done; NA, not available; −, negative; Ig, immunoglobulin; +, positive. †Lactate dehydrogenase 235; thick and thin blood smears for malaria negative; monoscreen negative; urine 2–5 erythrocytes, 100 protein; rapid respiratory panel negative. ‡Chest radiograph negative; HIV 1+2 antibody evaluation negative; *Clostridium difficile* testing negative; cryptosporidium and *Giardia* spp. immunoassays negative. §Chest radiograph negative; hepatitis B surface antibody negative; dengue IgG enzyme immunoassay negative; IgM enzyme immunoassay negative. ¶Sample from January 30 was PCR positive; samples from January 31 and February 1 were PCR negative.
